# The Treatment of Syphilis by Hypodermatic Injections, with Special Reference to the Salicylate of Mercury

**Published:** 1894-08

**Authors:** L. Amster

**Affiliations:** Atlanta, Ga.


### THE TREATMENT OF SYPHILIS BY HYPODERMATIC INJECTIONS, WITH SPECIAL REFERENCE TO THE SALICYLATE OF MERCURY.*

By L. Amster, M.D.,

Atlanta, Ga.

I do not intend to-night to direct your attention to any new discovery in medicine ; I do not even claim originality for the subject. After a careful study of the literature of syphilis, I find that the hypodermic treatment has been sadly neglected in this country, and almost entirely ignored by Southern physicians.

My attention was called to this subject about five years ago through a report of Professor Neuman, of Vienna, setting forth the advantages of the hypodermatic method with the salicylate of mercury. Acting upon his plan, I began this mode of treatment with very good success.

The favorable results obtained in two of my patients who came under the observation of Dr. E. L. Keyes, of New York, attracted his notice. In his letters to me he signified his intention to give the treatment a thorough test, and encouraged me in its further pursuance. As I understand since, Dr. Keyes regards the treatment with favor. It is my purpose to point out to you the advantages of the treatment of syphilis by the method of hypodermatic injection in general, and with the salicylate of mercury in particular.

Our enlightened age has ceased to consider syphilis an incurable disease. No syphilitic nowadays thinks of blowing out his brains as in olden times, or considers himself ostracized from society or the blessings of marriage. The knowledge and the treatment of this dreaded disease has made such rapid strides that to-day we place syphilis on the list of the most manageable diseases. Our only aim at present should be to emulate the motto of the divine Hippocrates in treating disease: to cure *cito, tuto et jucunde.*

In mercury we have the remedy *par excellence* in the treatment of syphilis. Syphilis and mercury are terms wedded to each other

* Read before the Atlanta Society of Medicine, May, 1894.

in the materia medica. If we therefore resort to the administration of mercury by means of hypodermatic injection, we possess a therapeutic agent which will accomplish our aim.

It only remains for us to select the most suitable and best preparation, and this I find to be the salicylate.

Before arguing the merits of this preparation, let us view the advantages of the hypodermatic treatment in general: Its first and foremost recommendation is extreme rapidity of action. External manifestations of syphilis can be made to disappear in an astonishingly short time. Patients of course desire to hide the disease, their sores and unsightly eruptions from their fellow creatures, and are gratified at the quick result. The physician during the whole course of treatment remains master of the situation. He is not obliged to rely upon the patient's statement that he has taken his medicine three times daily. Inject once a week and you are sure that the patient is under the influence of the drug. This is all the medicine necessary. The patient is spared the annoyance of taking medicine for a prolonged time. He is spared the large drug bills, and will volunteer a more liberal fee to his physician. The physician is certain of the purity of the drug, and can prepare the emulsion himself whenever required. The treatment saves time and labor, as it takes less time to inject once a week than to repeat instructions to patients, which are often not properly carried out. The digestive organs do not become affected, as is often the case in internal medication. No mercurial dermatitis occurs, as in the treatment by inunctions. It can be easily practiced where mercury is not borne well internally, and where inunctions are not practicable on account of the peculiar irritability of the skin. The moral effect produced upon the patient is good. He is impressed with the novelty of the treatment. He is relieved of the necessity of taking medicine and spared the annoyance of rubbing greasy, repulsive and ill-smelling salves into his body.

The disadvantages of the hypodermatic treatment are the pain and the abscesses produced at the seat of injection. The pain can be overcome to a great degree by the mode of injecting and the preparation employed. I have never met a patient yet who objected to the little pain and inconvenience of a hypodermatic after becoming aware of the advantages this mode of treatment offers. Abscesses will not be produced in a single case if the salicylate is employed and antiseptic precautions observed. Indurations at the point of injection do occasionally occur, but these are so slight as to often escape the notice of the patient, or they disappear in two or three days.

One other disadvantage which I have never had occasion to observe is mentioned by Blaschko in the *Revue de Therapeutique Medico-Chirurgicale.* Blaschko reports two cases in which the injections of mercury were followed by pulmonary symptoms. The patients complained of thoracic pain, cough, difficulty of respiration and bloody sputa. The symptoms in both cases disappeared in about three days. These symptoms are explained by Blaschko as being due to emboli caused by the paraffin employed as a vehicle for the suspension of the insoluble mercurial preparation. By using albolene or oil of vaseline these complications can be avoided, and the injections used less frequently in individuals affected with pulmonary troubles.

It is the universal opinion of all who have tried the hypodermatic treatment to any extent that the insoluble preparations of mercury are preferable to the soluble ones. The absorption of the insoluble preparations is gradual and complete, and the dose precise, keeping the patient in a state of chronic mecurialization. Insoluble preparations shorten the time of treatment considerably, are not so painful, and do not give rise to local abscesses.

There are two preparations which are unquestionably superior to any other salt of mercury—the sozoiodolate, the use of which must be necessarily limited on account of the considerable pain it causes, and the salicylate, which does away with all disagreeable features connected with the hypodermatic treatment of syphilis by other mercurial compounds.

Silva de Aranjo, of Rio de Janeiro, was one of the first to use the salicylate hypodermatically with excellent results. After him, Carl Szadek, of Kiev, administered this remedy obtaining similar good results. Oscar V. Peterson, of St. Petersburg, considers the salicylate the best insoluble compound for the hypodermatic treatment. In none of his forty consecutive cases treated by the salt, was there any pain complained of. Smirnoff and Scarenzio call this treatment "the ideal perfection in the therapeutics of syphilis." Tohiskiakoff, of St. Petersburg, tried the salicylate in one hundred and ninety-four patients, the sublimate in seventy, the cyanide in eleven, and decides in favor of the salicylate. Neuman, of Vienna, employed with satisfaction, the salicylate internally and hypodermatically. Theodore Schreus reports two hundred and nineteen cases, with one thousand five hundred and eighteen injections, with uniformly good results. Klotz, of New York, recommends the treatment very highly. Keyes regards the method and the preparation as satisfactory. In his last communication to me he says: "I prefer the salicylate of mercury to all other preparations I have ever employed, and I use it still whenever I have occasion to resort to hypodermatic medication." Blaschko believes mercurial injections give the best results in the treatment of syphilis. Endlitz, a man of rich experience, is decidedly in favor of the hypodermatic medication.

Contraindications to hypodermatic medication are:

Bright's disease, advanced anemia, general exhaustion, chronic alcoholism, infancy and delicate childhood.

The salicylate of mercury is a tasteless, odorless, white, amorphous, insoluble powder. It acts with great promptness and rapidity, causes little pain, does not produce suppuration, is gradually and completely absorbed, keeping the system under the influence of mercury without producing salivation or toxic symptoms, does not irritate the digestive organs, and hence is eminently efficacious in the treatment of syphilis by means of hypodermatic injections.

As a vehicle I have used paraffin, albolene and the oil of vaseline. Of these the oil of vaseline is the most satisfactory. Dr. Burlureaux claims sterilized oil to be the best vehicle for the suspension of insoluble mercurial preparations. He holds it to be even better than the liquid vaseline. It is more readily absorbed, and besides it is in itself a food of the first order, which can only be very beneficial in syphilis. I find the oil of vaseline quite satisfactory for ordinary purposes. The dose for a single hypodermatic injection is one grain of the salt to about twenty minims of oil of vaseline. The gluteal region is the most adapted for injection. One injection a week is sufficient, alternating from the right to the left side. Six to twelve injections are as a rule sufficient to eliminate every symptom of syphilis, the average number being nine.

Keyes thinks it a good plan, as he advised one of my patients, to continue the treatment with one or bi-weekly injections for the length of one year, notwithstanding all symptoms disappear, as no harm can be done by pursuing this course. Relapses occasionally occur, but less frequently than in any other method of treatment. The treatment is most effective in the primary and secondary stage. I have had little experience with it in tertiary syphilis, where we can hardly dispense with the iodides. Some observers claim good results with the hypodermatic method in the tertiary stage. It no doubt must be beneficial if administered in conjunction with the iodide internally. Cleanliness and antiseptic precautions are indispensable in the administration of hypodermatics.

Tieman, of New York, manufactures an antiseptic syringe expressly for the treatment of syphilis, but any ordinary hypodermatic syringe answers the purpose very well.

It is necessary to prepare the emulsion as thin as possible in order to make it pass through the hypodermatic needle. For that purpose weigh your powder, add a little of the oil of vaseline and triturate well, occasionally heating the mixture. Then add the rest of the oil, when the emulsion is ready for use.

The following is a good method of procedure: Wash the place to be injected, in the gluteal region, with a warm carbolized solution, wash the syringe and needle in the same carbolized solution. Warm the emulsion, fill your syringe and inject with a sharp needle deep into the gluteal muscles. After injecting, use gentle friction over the point of injection. Now wash your needle again in the carbolized solution, greese the needle with a little vaseline and insert the wire also greased. This latter precaution will counteract the tendency of the needle to become corroded from the mercury and you will be able to use the same needle for several months. Sometimes the patients complain of a feeling of heaviness and a little pain, especially upon retiring at night. A little opium or morphine will relieve this feeling and the pain

After the hypodermatic treatment is completed it is a good plan to place the patient upon a tonic.

There have been investigations carried on of late regarding the immunity of syphilis. Bonaduce has conducted a clinical study, based upon the supposition that after the contagious secondary period is past, there will be found in the blood and fluids of the body those chemical substances and antitoxines, which convey immunity. A patient with all typical symptoms of syphilis was injected with a serum prepared from the blood drawn from the placenta and children born with all characteristics of hereditary syphilis. In all, twelve injections were given. There was no inflammatory action excited. After the treatment the most minute search showed no sign of the disease.

Tomasoli and Fournier have obtained some results from injections with blood serum from animals naturally immune and supposed to contain the antitoxine of syphilis. These researches, which are at present conducted by many distinguished observers, introduce a novel feature in the therapeutics of syphilis; and if capable of replacing mercury and the iodides, it will cause a revolution in medicine equal to the epochs of Jenner, Pasteur and Koch.

In conclusion it would perhaps be proper to illustrate my statement with cases that come under my personal observation. This would hardly strengthen my plea however, and in view of the fact that I have already taxed your patience severely, I will simply repeat that I have obtained sufficiently good results from the treatment to warrant my appearing before you to plead for a favorable notice and a speedy trial of the hypodermatic treatment. I do not claim everything for the method, but I do emphatically state that in the majority of cases it is applicable. The hypodermatic treatment is no fad. It has passed the stage of experiment and has taken a well deserved place in therapeutics. I trust I have aroused some interest here to-night and that this field of investigation will receive more attention than heretofore. It is so easy to convince yourselves. The whole proceeding is so simple, that every liberal-minded physician should give it a trial.

[Shortly after the reading of this paper there appeared in the *Therapeutic Gazette* of May, a very thorough and able article by

Dr. L. Wolff, of Philadelphia, discussing the subject of hypodermatic medication of syphilis.

Dr. Wolff requested the opinion of some of the most prominent syphilographers in Continental Europe and America, regarding the treatment. He received forty-four answers, and of this number thirty-six use the hypodermatic treatment; the rest have not had sufficient experience with the method. They all concede that the treatment is established on a firm basis and that it will be continued as a regular treatment for syphilis. For the benefit of those who are ready to condemn the method without trial, I will quote from Dr. Wolff's article. "Condemnations without trial, or judging *a priori* without experience, can hardly be assumed as a fair means to arrive at medical opinions. . . . I think that it is one of the methods of treatments for syphilis which has a wide application and a vast field of usefulness."]

